# Supplementary material for: Use of Next Generation Sequencing to Define the Origin of Primary Myelofibrosis
Source: Cancers (Basel). 2023 Mar 15;15(6):1785. doi: 10.3390/cancers15061785 (PMC10046249; doi:10.3390/cancers15061785)
Supplement: Supplementary file 1 [file cancers-15-01785-s001.zip › cancers-2226809-supplementary.pdf]

Supplementary Table S1. Main clinical characteristics of patients enrolled as validation cohort.

Patient	Age (years)	BM fibrosis	Splenomegaly	Hb (g/dL)	WBC ($x10^9$/L)	PLTs ($x10^9$/L)
FI	71	II	grade II	9.6	10.57	126
PS	65	II	grade II	8.8	42	303
GG	75	II	grade II	9.2	4.7	152
CE	58	I	grade IV	12	15	150
CL	70	II	grade II	9.8	5.4	83
BV	81	I	grade II	12	13.5	196
VM	73	II	grade IV	8.7	26	170
DMFG	56	II	grade II	9.4	45	123
CGD	77	II	grade IV	9.5	10.5	398

Supplementary Table S2. General statistics for WES analysis

Sample	# Mate Pairs	% Mapped Reads (on total)	# base pair	Theoretical Coverage	Mean coverage	% of Exome covered at least 1X	% of Exome covered at least 10X	% base >Q30	Mean quality Score
GT_G	20.122.276	98,36%	4024455200	60X	31X	87,07%	76,93%	90,97	35,58
GT_L	15.862.059	98,35%	3172411800	48X	25X	86,94%	73,46%	90,87	35,55
GT_S	14.407.376	97,05%	2881475200	43X	24X	85,70%	71,17%	91,25	35,67
GB_G	10.306.336	98,11%	2061267200	31X	16X	86,39%	61,20%	94,37	36,64
GB_L	8.445.568	98,11%	1689113600	25X	14X	85,93%	52,12%	94,31	36,63
GB_S	7.025.907	96,24%	1405181400	21X	12X	85,60%	44,17%	94,56	36,69
CC_G	8.718.111	98,26%	1743622200	26X	14X	86,32%	52,37%	94,08	36,53
CC_L	7.754.762	98,30%	1550952400	23X	12X	85,94%	46,81%	94,15	36,59
CC_S	8.619.081	97,48%	1723816200	26X	14X	86,22%	54,18%	94,39	36,62

G: granulocytes
L: lymphocytes
S: saliva

Supplementary Table S3. SNVs recorded by WES

SAMPLES	# TOTAL SNV's called	# PASSED FILTER SNV's	Ti/Tv ratio	# SOMATIC SNV's	# EXONIC/SPLICING SNV's*	# NON SYNONIMUS SNV's	# NOVEL SNV's *
GT_G_L	415.579	126.369	1,82	45.969	3.173	1.540	80
GB_G_L	313.077	104.687	1,86	21.980	1.787	857	40
CC_G_L	282.391	102.479	1,94	17.077	1.283	603	26

*NOT IN DBSNP AND 1000GENOME

Supplementary Table S4. Novel SNVs

Chr	Start	End	Ref	Alt	Func.refGene	Gene.refGene	Recurrency/case	ExonicFunc.refGene	AAChange.refGene	cosmic64
5	140554571	140554571	C	T	exonic	*PCDHB7*	*1*	nonsynonymous SNV	PCDHB7:NM_018940:exon1:c.C2155T:p.P719S	ID=COSM370895;OCCURENCE=1(lung)
4	120107311	120107311	G	A	exonic	*MYOZ2*	*1*	nonsynonymous SNV	MYOZ2:NM_016599:exon6:c.G751A:p.E251K	ID=COSM202461;OCCURENCE=2(large_intestine)
2	202252470	202252470	C	T	exonic	*TRAK2*	*1*	nonsynonymous SNV	TRAK2:NM_015049:exon13:c.G1652A:p.R551Q	ID=COSM170962;OCCURENCE=1(large_intestine)
5	94852666	94852666	C	T	exonic	*TTC37*	*1*	nonsynonymous SNV	TTC37:NM_014639:exon22:c.G2390A:p.G797D	ID=COSM1230789;OCCURENCE=1(large_intestine)
3	195512042	195512042	T	C	exonic	*MUC4*	*2*	nonsynonymous SNV	MUC4:NM_018406:exon2:c.A6409G:p.T2137A	
3	195512042	195512042	T	C	exonic	*MUC4*	*2*	nonsynonymous SNV	MUC4:NM_018406:exon2:c.A6409G:p.T2137A	
2	219602830	219602830	G	A	exonic	*TTLL4*	*2*	nonsynonymous SNV	TTLL4:NM_014640:exon3:c.G431A:p.S144N	
2	219602830	219602830	G	A	exonic	*TTLL4*	*2*	nonsynonymous SNV	TTLL4:NM_014640:exon3:c.G431A:p.S144N	
20	37396220	37396220	C	G	exonic	*ACTR5*	*1*	nonsynonymous SNV	ACTR5:NM_024855:exon8:c.C1547G:p.P516R	
5	128983536	128983536	T	C	exonic	*ADAMTS19*	*1*	nonsynonymous SNV	ADAMTS19:NM_133638:exon12:c.T1933C:p.C645R	
15	89169891	89169891	A	T	exonic	*AEN*	*1*	nonsynonymous SNV	AEN:NM_022767:exon2:c.A451T:p.M151L	
16	56436922	56436922	G	A	exonic	*AMFR*	*1*	nonsynonymous SNV	AMFR:NM_001144:exon7:c.C949T:p.R317C	
11	57004348	57004348	G	A	exonic	*APLNR*	*1*	nonsynonymous SNV	APLNR:NM_005161:exon1:c.C131T:p.T44M	
3	153840224	153840224	G	C	exonic	*ARHGEF26*	*1*	nonsynonymous SNV	ARHGEF26:NM_001251962:exon2:c.G443C:p.R148P,ARHGEF26:	

									NM_001251963:exon2:c.G443C:p.R148P,ARHGEF26:NM_015595:exon2:c.G443C:p.R148P	
2	39185177	39185177	C	T	exonic	*ARHGEF33*	*1*	nonsynonymous SNV	ARHGEF33:NM_001145451:exon13:c.C1373T:p.S458L	
X	1553939	1553939	C	T	exonic	*ASMTL*	*1*	nonsynonymous SNV	ASMTL:NM_001173474:exon4:c.G328A:p.A110T,ASMTL:NM_001173473:exon5:c.G202A:p.A68T,ASMTL:NM_004192:exon5:c.G376A:p.A126T	
3	194167712	194167712	T	C	exonic	*ATP13A3*	*1*	nonsynonymous SNV	ATP13A3:NM_024524:exon13:c.A1441G:p.I481V	
16	28847485	28847485	G	A	exonic	*ATXN2L*	*1*	nonsynonymous SNV	ATXN2L:NM_007245:exon22:c.G3127A:p.D1043N,ATXN2L:NM_017492:exon22:c.G3127A:p.D1043N,ATXN2L:NM_145714:exon22:c.G3127A:p.D1043N,ATXN2L:NM_148414:exon22:c.G3127A:p.D1043N	
10	124050680	124050680	C	A	exonic	*BTBD16*	*1*	nonsynonymous SNV	BTBD16:NM_144587:exon7:c.C561A:p.H187Q	
10	124050606	124050606	G	T	exonic	*BTBD16*	*1*	nonsynonymous SNV	BTBD16:NM_144587:exon7:c.G487T:p.A163S	
14	59950650	59950650	G	C	exonic	*C14orf149*	*1*	nonsynonymous SNV	C14orf149:NM_144581:exon1:c.C385G:p.R129G	
15	40675158	40675158	C	T	exonic	*C15orf23*	*1*	nonsynonymous SNV	C15orf23:NM_001142761:exon1:c.C122T:p.A41V,C15orf23:NM_001142762:exon1:c.C122T:p.A41V,C15orf23:NM_033286:exon1:c.C122T:p.A41V	
19	757886	757886	C	T	exonic	*C19orf21*	*1*	nonsynonymous SNV	C19orf21:NM_173481:exon2:c.C940T:p.R314W	
19	1010785	1010785	G	A	exonic	*C19orf6*	*1*	nonsynonymous SNV	C19orf6:NM_001033026:exon11:c.C1427T:p.T476M	
21	47581946	47581946	G	C	exonic	*C21orf56*	*1*	nonsynonymous SNV	C21orf56:NM_032261:exon3:c.C118G:p.R40G,C21orf56:NM_001142854:exon4:c.C580G:p.R194G	
2	27804403	27804403	G	A	exonic	*C2orf16*	*1*	nonsynonymous SNV	C2orf16:NM_032266:exon1:c.G4964A:p.R1655H	

5	173416334	173416334	C	T	exonic	*C5orf47*	*1*	nonsynonymous SNV	C5orf47:NM_001144954:exon1:c.C68T:p.S23L	
17	4883619	4883619	T	C	exonic	*CAMTA2*	*1*	nonsynonymous SNV	CAMTA2:NM_001171166:exon8:c.A1004G:p.Q335R,CAMTA2:NM_001171168:exon8:c.A995G:p.Q332R,CAMTA2:NM_001171167:exon9:c.A1067G:p.Q356R,CAMTA2:NM_015099:exon9:c.A998G:p.Q333R	
12	7526150	7526150	T	C	exonic	*CD163L1*	*1*	nonsynonymous SNV	CD163L1:NM_174941:exon14:c.A3496G:p.S1166G	
22	17601344	17601344	A	G	exonic	*CECR6*	*1*	nonsynonymous SNV	CECR6:NM_031890:exon1:c.T674C:p.L225P	
22	46931137	46931137	T	G	exonic	*CELSR1*	*1*	nonsynonymous SNV	CELSR1:NM_014246:exon1:c.A1931C:p.E644A	
16	66997768	66997768	G	T	exonic	*CES3*	*1*	nonsynonymous SNV	CES3:NM_001185177:exon4:c.G490T:p.A164S,CES3:NM_024922:exon4:c.G490T:p.A164S	
2	189875078	189875078	A	G	exonic	*COL3A1*	*1*	nonsynonymous SNV	COL3A1:NM_000090:exon49:c.A3998G:p.D1333G	
10	99683140	99683140	C	T	exonic	*CRTAC1*	*1*	nonsynonymous SNV	CRTAC1:NM_001206528:exon4:c.G439A:p.D147N,CRTAC1:NM_018058:exon4:c.G439A:p.D147N	
10	16942708	16942708	A	G	exonic	*CUBN*	*1*	nonsynonymous SNV	CUBN:NM_001081:exon53:c.T8326C:p.S2776P	
14	24589085	24589085	A	C	exonic	*DCAF11*	*1*	nonsynonymous SNV	DCAF11:NM_001163484:exon11:c.A1072C:p.I358L,DCAF11:NM_025230:exon11:c.A1072C:p.I358L,DCAF11:NM_181357:exon11:c.A994C:p.I332L	
12	132625856	132625856	A	G	exonic	*DDX51*	*1*	nonsynonymous SNV	DDX51:NM_175066:exon8:c.T1214C:p.L405P	
9	126433616	126433616	T	C	exonic	*DENND1A*	*1*	nonsynonymous SNV	DENND1A:NM_020946:exon7:c.A407G:p.H136R,DENND1A:NM_024820:exon7:c.A407G:p.H136R	
1	153915373	153915373	C	T	exonic	*DENND4B*	*1*	nonsynonymous SNV	DENND4B:NM_014856:exon3:c.G551A:p.R184H	
8	145541770	145541770	G	T	exonic	*DGAT1*	*1*	nonsynonymous SNV	DGAT1:NM_012079:exon8:c.C739A:p.L247M	

8	12957206	12957206	G	T	exonic	*DLC1*	*1*	nonsynonymous SNV	DLC1:NM_001164271:exon5:c.C1107A:p.D369E,DLC1:NM_006094:exon5:c.C1329A:p.D443E,DLC1:NM_182643:exon9:c.C2640A:p.D880E	
3	57457297	57457297	G	C	exonic	*DNAH12*	*1*	nonsynonymous SNV	DNAH12:NM_178504:exon15:c.C1813G:p.L605V	
6	56365932	56365932	C	A	exonic	*DST*	*1*	nonsynonymous SNV	DST:NM_015548:exon61:c.G11973T:p.Q3991H	
1	167095444	167095444	G	A	exonic	*DUSP27*	*1*	nonsynonymous SNV	DUSP27:NM_001080426:exon5:c.G1076A:p.G359D	
20	2730050	2730050	G	A	exonic	*EBF4*	*1*	nonsynonymous SNV	EBF4:NM_001110514:exon9:c.G631A:p.V211M	
2	212251646	212251646	T	C	exonic	*ERBB4*	*1*	nonsynonymous SNV	ERBB4:NM_001042599:exon26:c.A3365G:p.E1122G,ERBB4:NM_005235:exon27:c.A3413G:p.E1138G	
11	124626602	124626602	G	A	exonic	*ESAM*	*1*	nonsynonymous SNV	ESAM:NM_138961:exon3:c.C286T:p.P96S	
16	68265180	68265180	C	T	exonic	*ESRP2*	*1*	nonsynonymous SNV	ESRP2:NM_024939:exon12:c.G1612A:p.V538M	
1	32713997	32713997	A	G	exonic	*FAM167B*	*1*	nonsynonymous SNV	FAM167B:NM_032648:exon2:c.A287G:p.Q96R	
7	208930	208930	A	G	exonic	*FAM20C*	*1*	nonsynonymous SNV	FAM20C:NM_020223:exon3:c.A817G:p.I273V	
22	24982231	24982231	C	G	exonic	*FAM211B*	*1*	nonsynonymous SNV	FAM211B:NM_207644:exon4:c.G571C:p.G191R	
1	11733831	11733831	G	A	exonic	*FBXO6*	*1*	nonsynonymous SNV	FBXO6:NM_018438:exon6:c.G805A:p.E269K	
12	4554391	4554391	T	C	exonic	*FGF6*	*1*	nonsynonymous SNV	FGF6:NM_020996:exon1:c.A346G:p.S116G	
10	123263368	123263368	T	C	exonic	*FGFR2*	*1*	nonsynonymous SNV	FGFR2:NM_001144914:exon7:c.A1039G:p.M347V,FGFR2:NM_001144916:exon7:c.A1030G:p.M344V,FGFR2:NM_001144917:exon8:c.A1027G:p.M343V,FGFR2:NM_001144918:exon8:c.A1024G:p.M342V,FGFR2:NM_001144913:exon9:c.A1378G:p.M460V,FGFR2:NM_001144915:exon9:c.A1108G:p	

									.M370V,FGFR2:NM_001144919:exon9:c.A1111G:p.M371V,FGFR2:NM_000141:exon10:c.A1375G:p.M459V,FGFR2:NM_022970:exon10:c.A1378G:p.M460V	
17	74133451	74133451	C	T	exonic	*FOXJ1*	*1*	nonsynonymous SNV	FOXJ1:NM_001454:exon3:c.G1249A:p.V417M	
4	48636373	48636373	T	G	exonic	*FRYL*	*1*	nonsynonymous SNV	FRYL:NM_015030:exon4:c.A55C:p.S19R	
6	52993344	52993344	G	A	exonic	*GCM1*	*1*	nonsynonymous SNV	GCM1:NM_003643:exon6:c.C971T:p.P324L	
1	228346699	228346699	C	G	exonic	*GJC2*	*1*	nonsynonymous SNV	GJC2:NM_020435:exon2:c.C1240G:p.R414G	
16	4385106	4385106	A	G	exonic	*GLIS2*	*1*	nonsynonymous SNV	GLIS2:NM_032575:exon4:c.A568G:p.N190D	
12	110906419	110906419	G	T	exonic	*GPN3*	*1*	nonsynonymous SNV	GPN3:NM_001164372:exon1:c.C23A:p.A8D	
12	6934685	6934685	C	T	exonic	*GPR162*	*1*	nonsynonymous SNV	GPR162:NM_014449:exon3:c.C52T:p.P18S,GPR162:NM_019858:exon3:c.C904T:p.P302S	
7	73929830	73929830	G	A	exonic	*GTF2IRD1*	*1*	nonsynonymous SNV	GTF2IRD1:NM_001199207:exon4:c.G421A:p.G141S,GTF2IRD1:NM_005685:exon4:c.G325A:p.G109S,GTF2IRD1:NM_016328:exon4:c.G325A:p.G109S	
8	145223339	145223339	A	G	exonic	*HEATR7A*	*1*	nonsynonymous SNV	HEATR7A:NM_001099281:exon3:c.A164G:p.D55G,HEATR7A:NM_001099280:exon4:c.A164G:p.D55G,HEATR7A:NM_032450:exon4:c.A164G:p.D55G	
19	55777263	55777263	G	A	exonic	*HSPBP1*	*1*	nonsynonymous SNV	HSPBP1:NM_012267:exon6:c.C884T:p.A295V,HSPBP1:NM_001130106:exon7:c.C884T:p.A295V	
16	70883818	70883818	G	T	exonic	*HYDIN*	*1*	nonsynonymous SNV	HYDIN:NM_032821:exon75:c.C12681A:p.F4227L	
8	39775642	39775642	C	G	exonic	*IDO1*	*1*	nonsynonymous SNV	IDO1:NM_002164:exon3:c.C219G:p.H73Q	
1	158986408	158986408	G	C	exonic	*IFI16*	*1*	nonsynonymous SNV	IFI16:NM_005531:exon4:c.G467C:p.G156A	

2	27695128	27695128	G	A	exonic	*IFT172*	*1*	nonsynonymous SNV	IFT172:NM_015662:exon15:c.C1513T:p.R505W	
15	65627092	65627092	C	T	exonic	*IGDCC3*	*1*	nonsynonymous SNV	IGDCC3:NM_004884:exon5:c.G797A:p.R266H	
16	87723830	87723830	G	A	exonic	*JPH3*	*1*	nonsynonymous SNV	JPH3:NM_020655:exon4:c.G1864A:p.E622K	
14	88729737	88729737	C	T	exonic	*KCNK10*	*1*	nonsynonymous SNV	KCNK10:NM_021161:exon2:c.G196A:p.V66I,KCNK10:NM_138317:exon2:c.G211A:p.V71I,KCNK10:NM_138318:exon2:c.G211A:p.V71I	
14	105359981	105359981	C	T	exonic	*KIAA0284*	*1*	nonsynonymous SNV	KIAA0284:NM_015005:exon14:c.C4055T:p.T1352M,KIAA0284:NM_001112726:exon15:c.C4160T:p.T1387M	
6	138657595	138657595	T	G	exonic	*KIAA1244*	*1*	nonsynonymous SNV	KIAA1244:NM_020340:exon34:c.T6506G:p.V2169G	
20	60889715	60889715	C	T	exonic	*LAMA5*	*1*	nonsynonymous SNV	LAMA5:NM_005560:exon61:c.G8263A:p.D2755N	
4	1827316	1827316	T	C	exonic	*LETM1*	*1*	nonsynonymous SNV	LETM1:NM_012318:exon7:c.A1175G:p.D392G	
15	101523754	101523754	G	A	exonic	*LRRK1*	*1*	nonsynonymous SNV	LRRK1:NM_024652:exon4:c.G283A:p.A95T	
11	63933390	63933390	G	A	exonic	*MACROD1*	*1*	nonsynonymous SNV	MACROD1:NM_014067:exon1:c.C77T:p.P26L	
6	119252877	119252877	A	C	exonic	*MCM9*	*1*	nonsynonymous SNV	MCM9:NM_017696:exon1:c.T12G:p.D4E,MCM9:NM_153255:exon2:c.T12G:p.D4E	
19	50321621	50321621	C	A	exonic	*MED25*	*1*	nonsynonymous SNV	MED25:NM_030973:exon1:c.C23A:p.P8Q	
19	36224316	36224316	C	T	exonic	*MLL4*	*1*	nonsynonymous SNV	MLL4:NM_014727:exon28:c.C6866T:p.P2289L	
20	49575899	49575899	G	T	exonic	*MOCS3*	*1*	nonsynonymous SNV	MOCS3:NM_014484:exon1:c.G520T:p.D174Y	
2	71360666	71360666	A	T	exonic	*MPHOSPH10*	*1*	nonsynonymous SNV	MPHOSPH10:NM_005791:exon2:c.A728T:p.D243V	
10	28409147	28409147	A	C	exonic	*MPP7*	*1*	nonsynonymous SNV	MPP7:NM_173496:exon12:c.T863G:p.I288S	
22	30409517	30409517	A	G	exonic	*MTMR3*	*1*	nonsynonymous SNV	MTMR3:NM_021090:exon14:c.A1490G:p.N497S,MTMR3:NM_1530	

									50:exon14:c.A1490G:p.N497S,MTMR3:NM_153051:exon14:c.A1490G:p.N497S	
7	100692168	100692168	C	T	exonic	*MUC17*	*1*	nonsynonymous SNV	MUC17:NM_001040105:exon5:c.C12578T:p.T4193I	
10	26443678	26443678	G	A	exonic	*MYO3A*	*1*	nonsynonymous SNV	MYO3A:NM_017433:exon25:c.G2719A:p.D907N	
10	51586619	51586619	A	G	exonic	*NCOA4*	*1*	nonsynonymous SNV	NCOA4:NM_001145260:exon11:c.A1897G:p.K633E	
8	24813744	24813744	C	T	exonic	*NEFL*	*1*	nonsynonymous SNV	NEFL:NM_006158:exon1:c.G286A:p.D96N	
11	284304	284304	C	T	exonic	*NLRP6*	*1*	nonsynonymous SNV	NLRP6:NM_138329:exon6:c.C2276T:p.T759M	
1	11907244	11907244	G	A	exonic	*NPPA*	*1*	nonsynonymous SNV	NPPA:NM_006172:exon2:c.C376T:p.R126W	
2	27656546	27656546	C	G	exonic	*NRBP1*	*1*	nonsynonymous SNV	NRBP1:NM_013392:exon3:c.C217G:p.Q73E	
12	2944085	2944085	G	A	exonic	*NRIP2*	*1*	nonsynonymous SNV	NRIP2:NM_031474:exon1:c.C65T:p.T22M	
1	228566324	228566324	G	T	exonic	*OBSCN*	*1*	nonsynonymous SNV	OBSCN:NM_001098623:exon105:c.G23735T:p.R7912L	
1	228402583	228402583	C	A	exonic	*OBSCN*	*1*	nonsynonymous SNV	OBSCN:NM_001098623:exon5:c.C1612A:p.H538N,OBSCN:NM_052843:exon5:c.C1612A:p.H538N	
13	53617310	53617310	G	A	exonic	*OLFM4*	*1*	nonsynonymous SNV	OLFM4:NM_006418:exon4:c.G641A:p.R214Q	
11	6221100	6221100	T	C	exonic	*OR52W1*	*1*	nonsynonymous SNV	OR52W1:NM_001005178:exon1:c.T647C:p.I216T	
8	107726058	107726058	C	G	exonic	*OXR1*	*1*	nonsynonymous SNV	OXR1:NM_181354:exon9:c.C1780G:p.H594D,OXR1:NM_001198532:exon10:c.C1804G:p.H602D,OXR1:NM_001198533:exon11:c.C1801G:p.H601D,OXR1:NM_018002:exon11:c.C1801G:p.H601D	
11	74053620	74053620	C	G	exonic	*PGM2L1*	*1*	nonsynonymous SNV	PGM2L1:NM_173582:exon12:c.G1518C:p.R506S	
3	17056251	17056251	C	G	exonic	*PLCL2*	*1*	nonsynonymous SNV	PLCL2:NM_015184:exon3:c.C2488G:p.L830V	
22	50714165	50714165	C	T	exonic	*PLXNB2*	*1*	nonsynonymous SNV	PLXNB2:NM_012401:exon37:c.G5485A:p.A1829T	

19	43258598	43258598	T	G	exonic	*PSG8*	*1*	nonsynonymous SNV	PSG8:NM_001130168:exon4:c.A764C:p.Q255P,PSG8:NM_001130167:exon5:c.A1130C:p.Q377P,PSG8:NM_182707:exon5:c.A1130C:p.Q377P	
20	48129726	48129726	G	A	exonic	*PTGIS*	*1*	nonsynonymous SNV	PTGIS:NM_000961:exon8:c.C1097T:p.A366V	
1	8421196	8421196	G	A	exonic	*RERE*	*1*	nonsynonymous SNV	RERE:NM_001042682:exon8:c.C709T:p.P237S,RERE:NM_001042681:exon18:c.C2371T:p.P791S,RERE:NM_012102:exon19:c.C2371T:p.P791S	
1	114354575	114354575	C	A	exonic	*RSBN1*	*1*	nonsynonymous SNV	RSBN1:NM_018364:exon1:c.G460T:p.A154S	
21	43896025	43896025	C	T	exonic	*RSPH1*	*1*	nonsynonymous SNV	RSPH1:NM_080860:exon8:c.G860A:p.R287H	
9	139298597	139298597	T	G	exonic	*SDCCAG3*	*1*	nonsynonymous SNV	SDCCAG3:NM_001039708:exon7:c.A899C:p.Q300P,SDCCAG3:NM_006643:exon8:c.A1049C:p.Q350P,SDCCAG3:NM_001039707:exon9:c.A1118C:p.Q373P	
22	30891372	30891372	C	T	exonic	*SEC14L4*	*1*	nonsynonymous SNV	SEC14L4:NM_001161368:exon5:c.G292A:p.V98M,SEC14L4:NM_174977:exon5:c.G292A:p.V98M	
9	139358954	139358954	C	A	exonic	*SEC16A*	*1*	nonsynonymous SNV	SEC16A:NM_014866:exon9:c.G4364T:p.G1455V	
1	156146634	156146634	G	A	exonic	*SEMA4A*	*1*	nonsynonymous SNV	SEMA4A:NM_001193302:exon13:c.G1736A:p.R579Q,SEMA4A:NM_001193300:exon15:c.G2132A:p.R711Q,SEMA4A:NM_001193301:exon15:c.G2132A:p.R711Q,SEMA4A:NM_022367:exon15:c.G2132A:p.R711Q	
15	48063897	48063897	C	T	exonic	*SEMA6D*	*1*	nonsynonymous SNV	SEMA6D:NM_020858:exon17:c.C2951T:p.P984L,SEMA6D:NM_153616:exon17:c.C2912T:p.P971L,SEMA6D:NM_153617:exon18:c.C2969T:p.P990L,SEMA6D:NM_153618:exon19:c.C3137T:p.P1046L	

									,SEMA6D:NM_001198999:exon20:c.C2951T:p.P984L	
4	8230069	8230069	C	A	exonic	*SH3TC1*	*1*	nonsynonymous SNV	SH3TC1:NM_018986:exon12:c.C2648A:p.A883D	
1	160616666	160616666	C	T	exonic	*SLAMF1*	*1*	nonsynonymous SNV	SLAMF1:NM_003037:exon1:c.G70A:p.G24R	
8	82606054	82606054	T	C	exonic	*SLC10A5*	*1*	nonsynonymous SNV	SLC10A5:NM_001010893:exon1:c.A1154G:p.Q385R	
15	85478705	85478705	G	A	exonic	*SLC28A1*	*1*	nonsynonymous SNV	SLC28A1:NM_004213:exon15:c.G1537A:p.A513T	
8	22273450	22273450	G	A	exonic	*SLC39A14*	*1*	nonsynonymous SNV	SLC39A14:NM_001128431:exon6:c.G919A:p.V307M,SLC39A14:NM_001135153:exon6:c.G919A:p.V307M,SLC39A14:NM_001135154:exon6:c.G919A:p.V307M,SLC39A14:NM_015359:exon6:c.G919A:p.V307M	
12	346452	346452	G	A	exonic	*SLC6A13*	*1*	nonsynonymous SNV	SLC6A13:NM_001190997:exon4:c.C292T:p.R98W,SLC6A13:NM_016615:exon6:c.C568T:p.R190W	
17	1687713	1687713	A	T	exonic	*SMYD4*	*1*	nonsynonymous SNV	SMYD4:NM_052928:exon8:c.T1927A:p.S643T	
4	52927035	52927035	C	G	exonic	*SPATA18*	*1*	nonsynonymous SNV	SPATA18:NM_145263:exon3:c.C281G:p.S94C	
15	91769810	91769810	G	A	exonic	*SV2B*	*1*	nonsynonymous SNV	SV2B:NM_014848:exon3:c.G317A:p.R106H	
16	29998831	29998831	G	C	exonic	*TAOK2*	*1*	nonsynonymous SNV	TAOK2:NM_016151:exon16:c.G3238C:p.G1080R,TAOK2:NM_001252043:exon17:c.G2899C:p.G967R	
4	107016790	107016790	C	G	exonic	*TBCK*	*1*	nonsynonymous SNV	TBCK:NM_033115:exon23:c.G2231C:p.R744P,TBCK:NM_001163435:exon25:c.G2420C:p.R807P,TBCK:NM_001163436:exon25:c.G2420C:p.R807P,TBCK:NM_001163437:exon25:c.G2303C:p.R768P	
14	90437568	90437568	C	T	exonic	*TDP1*	*1*	nonsynonymous SNV	TDP1:NM_001008744:exon5:c.C709T:p.H237Y,TDP1:NM_018319:exon6:c.C709T:p.H237Y	

9	103070806	103070806	C	T	exonic	*TEX10*	*1*	nonsynonymous SNV	TEX10:NM_001161584:exon13:c.G2450A:p.G817E,TEX10:NM_017746:exon13:c.G2441A:p.G814E	
17	32964871	32964871	G	A	exonic	*TMEM132E*	*1*	nonsynonymous SNV	TMEM132E:NM_207313:exon10:c.G2575A:p.E859K	
19	16793274	16793274	G	A	exonic	*TMEM38A*	*1*	nonsynonymous SNV	TMEM38A:NM_024074:exon4:c.G509A:p.R170Q	
1	1149477	1149477	C	T	exonic	*TNFRSF4*	*1*	nonsynonymous SNV	TNFRSF4:NM_003327:exon1:c.G31A:p.G11R	
6	167753708	167753708	G	T	exonic	*TTLL2*	*1*	nonsynonymous SNV	TTLL2:NM_031949:exon3:c.G320T:p.G107V	
2	179428520	179428520	C	T	exonic	*TTN*	*1*	nonsynonymous SNV	TTN:NM_003319:exon154:c.G55144A:p.V18382M,TTN:NM_133432:exon155:c.G55519A:p.V18507M,TTN:NM_133437:exon155:c.G55720A:p.V18574M,TTN:NM_133378:exon275:c.G74635A:p.V24879M,TTN:NM_001256850:exon276:c.G77416A:p.V25806M	
19	34942973	34942973	G	A	exonic	*UBA2*	*1*	nonsynonymous SNV	UBA2:NM_005499:exon10:c.G959A:p.R320H	
1	229772758	229772758	C	G	exonic	*URB2*	*1*	nonsynonymous SNV	URB2:NM_014777:exon4:c.C2398G:p.L800V	
4	85600104	85600104	A	G	exonic	*WDFY3*	*1*	nonsynonymous SNV	WDFY3:NM_014991:exon65:c.T10115C:p.I3372T	
7	158694446	158694446	A	G	exonic	*WDR60*	*1*	nonsynonymous SNV	WDR60:NM_018051:exon9:c.A1076G:p.E359G	
8	10755623	10755623	T	C	exonic	*XKR6*	*1*	nonsynonymous SNV	XKR6:NM_173683:exon3:c.A1765G:p.M589V	
6	43496622	43496622	G	A	exonic	*XPO5*	*1*	nonsynonymous SNV	XPO5:NM_020750:exon24:c.C2719T:p.H907Y	
16	11868187	11868187	T	C	exonic	*ZC3H7A*	*1*	nonsynonymous SNV	ZC3H7A:NM_014153:exon9:c.A808G:p.M270V	
19	44681693	44681693	T	A	exonic	*ZNF226*	*1*	nonsynonymous SNV	ZNF226:NM_001032372:exon6:c.T2278A:p.C760S,ZNF226:NM_001032373:exon6:c.T2278A:p.C760S	
7	64389235	64389235	G	A	exonic	*ZNF273*	*1*	nonsynonymous SNV	ZNF273:NM_021148:exon4:c.G1529A:p.G510E	

12	54764432	54764432	G	T	exonic	*ZNF385A*	*1*	nonsynonymous SNV	ZNF385A:NM_001130968:exon6:c.C665A:p.S222Y,ZNF385A:NM_015481:exon6:c.C848A:p.S283Y,ZNF385A:NM_001130967:exon7:c.C908A:p.S303Y	
16	3434804	3434804	G	C	exonic	*ZNF434*	*1*	nonsynonymous SNV	ZNF434:NM_017810:exon5:c.C253G:p.Q85E	
19	58991699	58991699	C	T	exonic	*ZNF446*	*1*	nonsynonymous SNV	ZNF446:NM_017908:exon7:c.C959T:p.T320M	
19	57909975	57909975	C	T	exonic	*ZNF548*	*1*	nonsynonymous SNV	ZNF548:NM_152909:exon3:c.C320T:p.P107L,ZNF548:NM_001172773:exon4:c.C356T:p.P119L	
12	133633570	133633570	G	T	exonic	*ZNF84*	*1*	nonsynonymous SNV	ZNF84:NM_001127372:exon5:c.G269T:p.W90L,ZNF84:NM_003428:exon5:c.G269T:p.W90L	
8	105463484	105463484	C	T	exonic	*DPYS*	*1*	stopgain SNV	DPYS:NM_001385:exon2:c.G413A:p.W138X	
1	11129712	11129712	G	A	exonic	*EXOSC10*	*1*	stopgain SNV	EXOSC10:NM_002685:exon21:c.C2317T:p.R773X,EXOSC10:NM_001001998:exon22:c.C2392T:p.R798X	
17	39884028	39884028	G	A	exonic	*HAP1*	*1*	stopgain SNV	HAP1:NM_177977:exon8:c.C1261T:p.Q421X	
22	25131820	25131820	T	A	exonic	*PIWIL3*	*1*	stopgain SNV	PIWIL3:NM_001008496:exon13:c.A1489T:p.R497X,PIWIL3:NM_001255975:exon13:c.A1489T:p.R497X	

Supplementary Figure S1. Occurrence of novel SNVs (A) and novel InDels (B). The X-axis graphs the number of cases. The Y-axis displays the chromosomes.

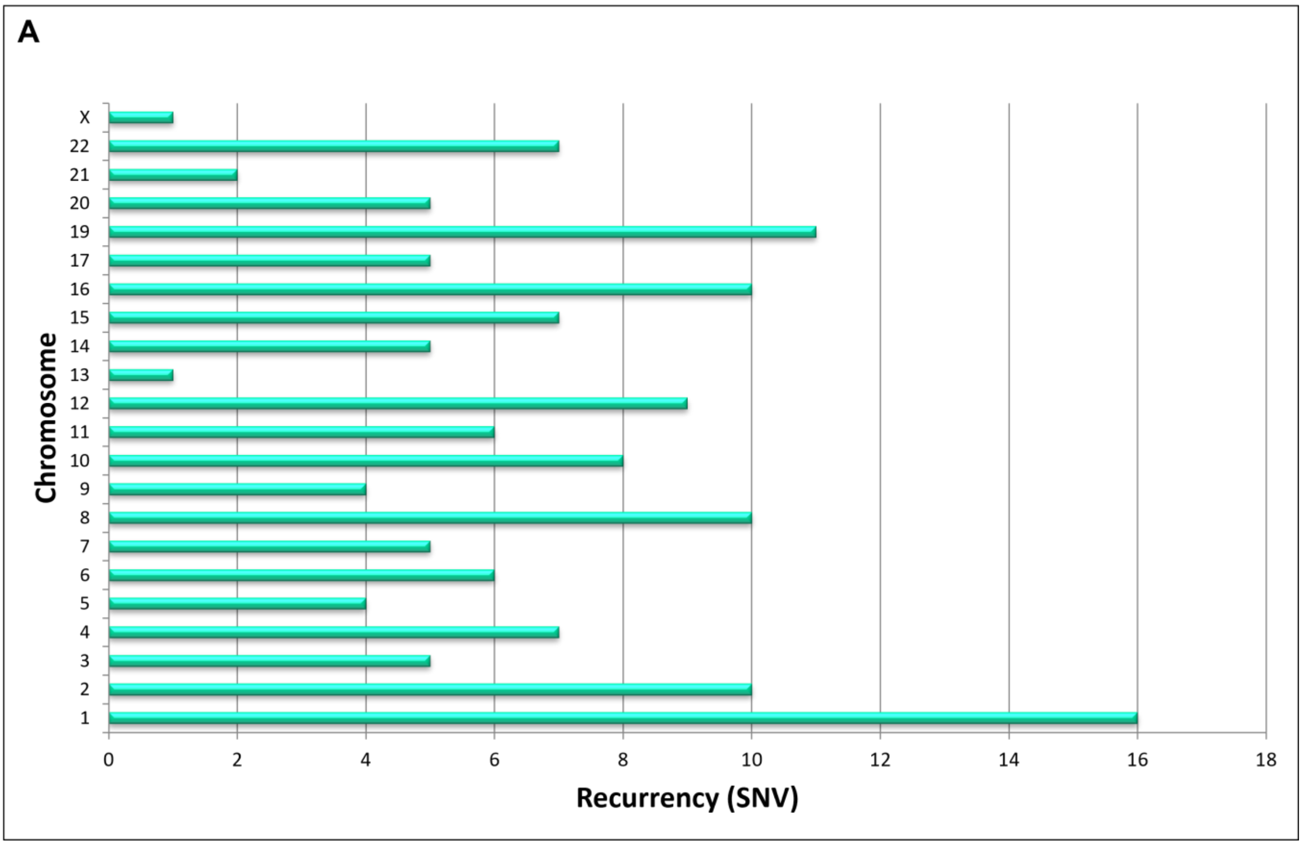

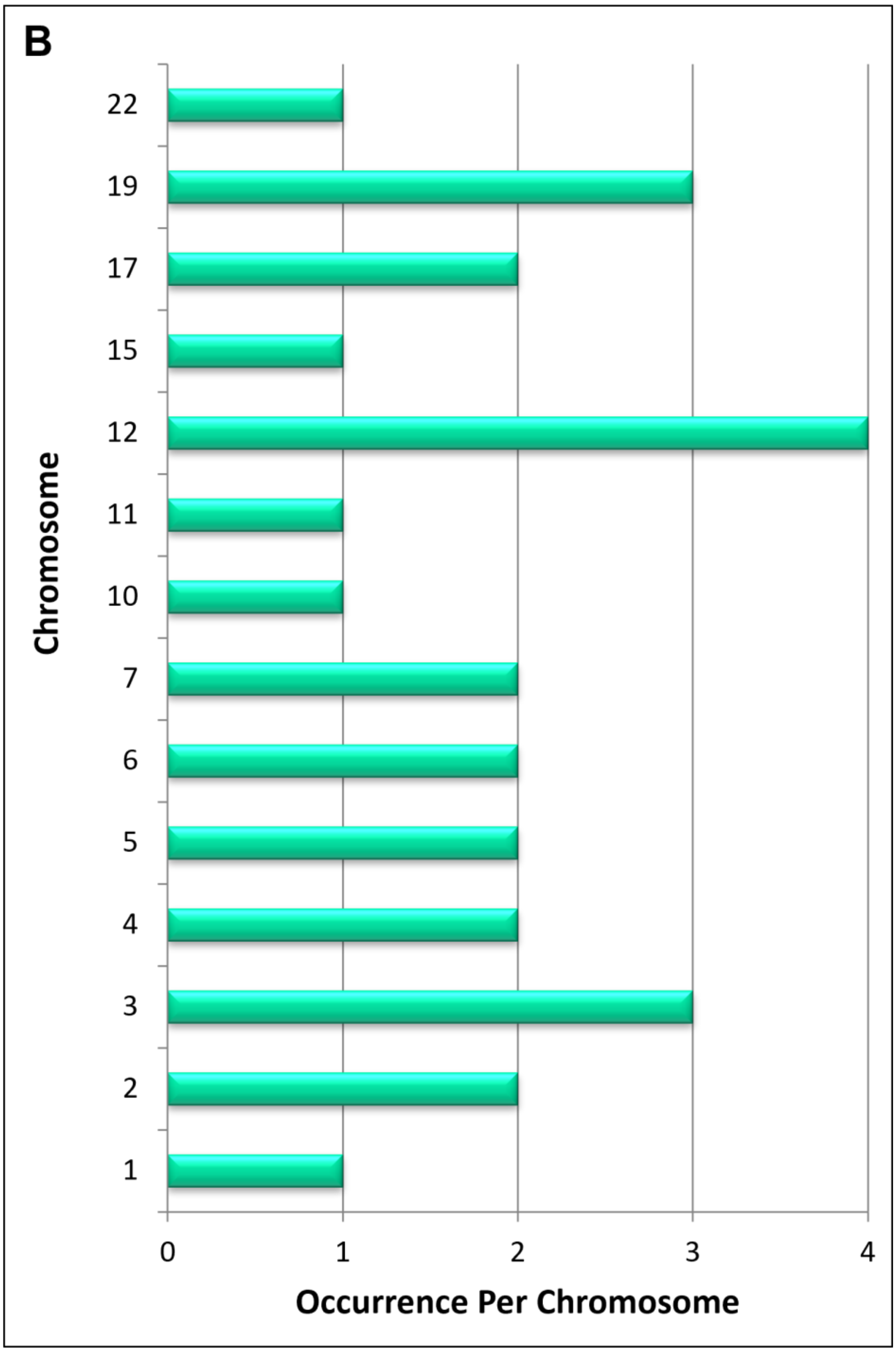
B
Chromosome
22
19
17
15
12
11
10
7
6
5
4
3
2
1
0
1
2
3
4
Occurrence Per Chromosome

Supplementary Figure S2. Functional analysis of genes affected by SNVs. The affected genes were analyzed in terms of enrichment of Gene Ontology Biological Processes (A). Pathways analysis aimed to recognize actionable genes was performed by Cognoscente (B).

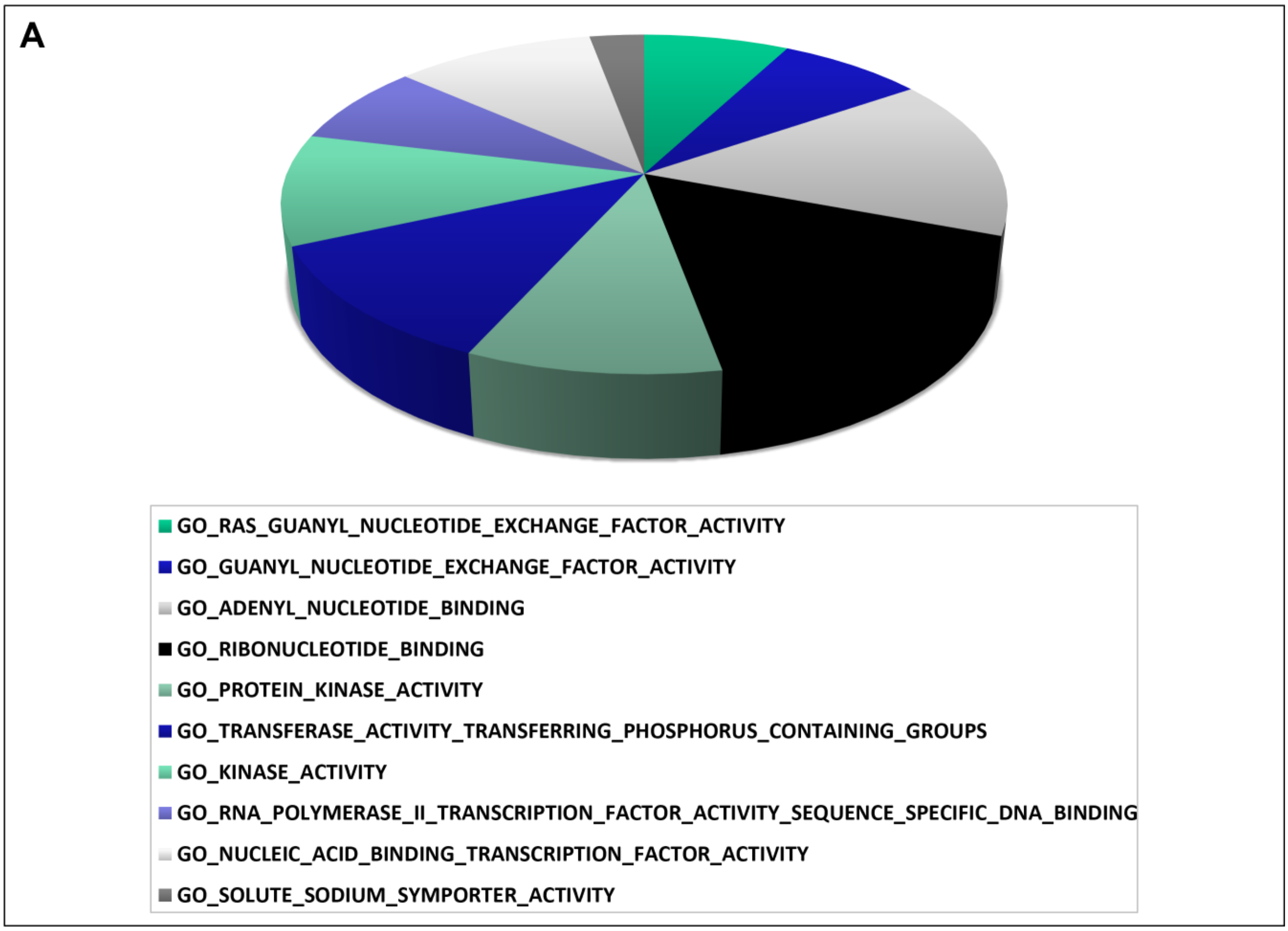

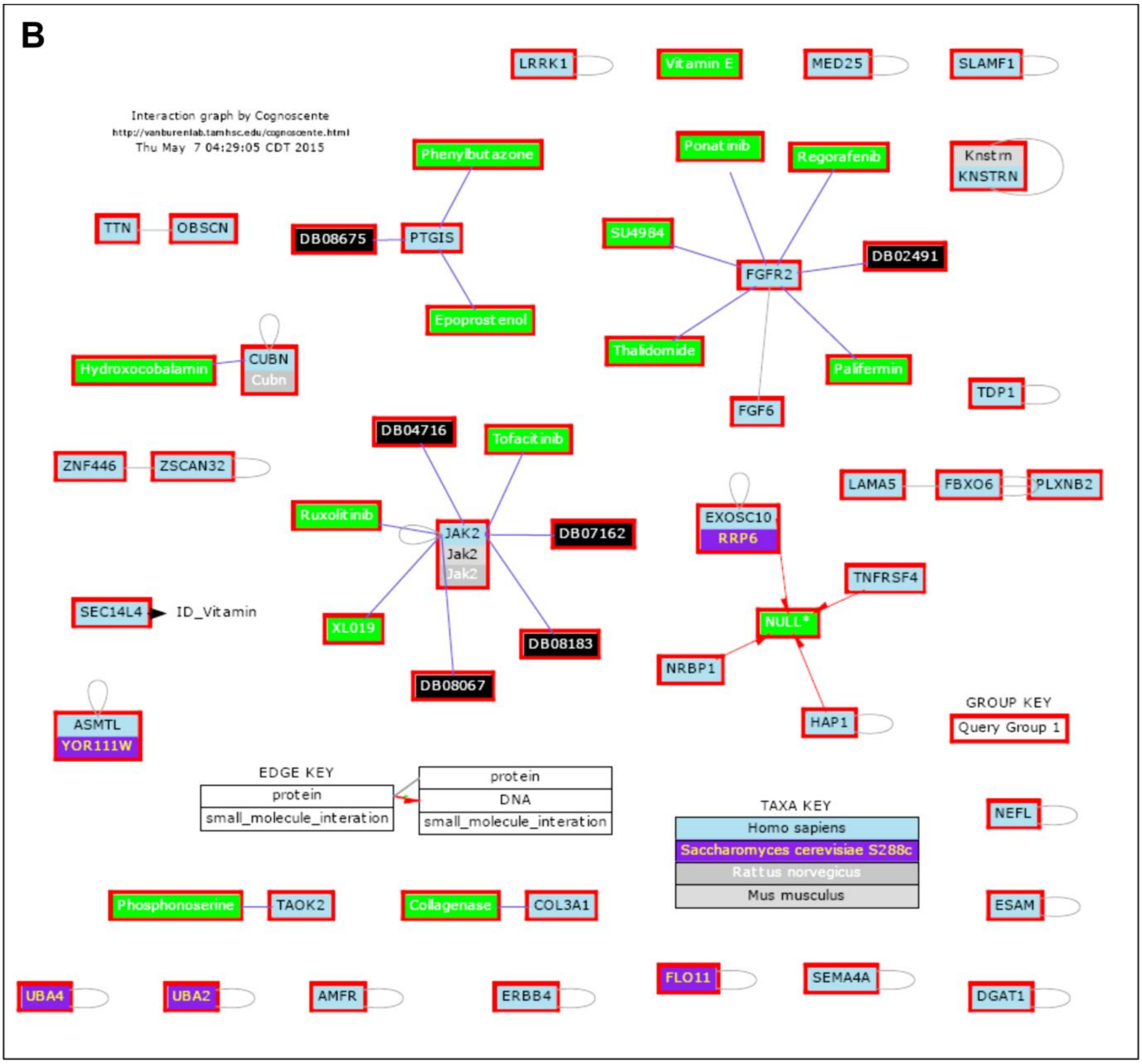
B
Interaction graph by Cognoscente
http://vanburenlab.tamhsc.edu/cognoscente.html
Thu May 7 04:29:05 CDT 2015
LRRK1
Vitamin E
MED25
SLAMF1
Phenylbutazone
Ponatinib
Regorafenib
Knstrn
KNSTRN
TTN
OBSCN
DB08675
PTGIS
SU4984
FGFR2
DB02491
Epoprostenol
Thalidomide
Palifermin
Hydroxocobalamin
CUBN
Cubn
FGF6
TDP1
DB04716
Tofacitinib
ZNF446
ZSCAN32
LAMA5
FBXO6
PLXNB2
Ruxolitinib
JAK2
Jak2
Jak2
DB07162
EXOSC10
RRP6
TNFRSF4
SEC14L4
ID_Vitamin
XL019
DB08183
NULL*
NRBP1
DB08067
ASMTL
YOR111W
HAP1
GROUP KEY
Query Group 1
EDGE KEY
protein
small_molecule_interation
protein
DNA
small_molecule_interation
TAXA KEY
Homo sapiens
Saccharomyces cerevisiae S288c
Rattus norvegicus
Mus musculus
NEFL
Phosphonoserine
TAOK2
Collagenase
COL3A1
ESAM
UBA4
UBA2
AMFR
ERBB4
FLO11
SEMA4A
DGAT1
